# Studentized bootstrap model-averaged tail area intervals

**DOI:** 10.1371/journal.pone.0213715

**Published:** 2019-03-18

**Authors:** Jiaxu Zeng, David Fletcher, Peter W. Dillingham, Christopher E. Cornwall

**Affiliations:** 1 Department of Preventive and Social Medicine, University of Otago, Dunedin, New Zealand; 2 Department of Mathematics and Statistics, University of Otago, Dunedin, New Zealand; 3 School of Science and Technology, University of New England, Armidale, Australia; 4 School of Biological Sciences, Victoria University of Wellington, Wellington, New Zealand; University of Illinois at Chicago College of Medicine, UNITED STATES

## Abstract

In many scientific studies, the underlying data-generating process is unknown and multiple statistical models are considered to describe it. For example, in a factorial experiment we might consider models involving just main effects, as well as those that include interactions. Model-averaging is a commonly-used statistical technique to allow for model uncertainty in parameter estimation. In the frequentist setting, the model-averaged estimate of a parameter is a weighted mean of the estimates from the individual models, with the weights typically being based on an information criterion, cross-validation, or bootstrapping. One approach to building a model-averaged confidence interval is to use a Wald interval, based on the model-averaged estimate and its standard error. This has been the default method in many application areas, particularly those in the life sciences. The MA-Wald interval, however, assumes that the studentized model-averaged estimate has a normal distribution, which can be far from true in practice due to the random, data-driven model weights. Recently, the model-averaged tail area Wald interval (MATA-Wald) has been proposed as an alternative to the MA-Wald interval, which only assumes that the studentized estimate from each model has a *N*(0, 1) or *t*-distribution, when that model is true. This alternative to the MA-Wald interval has been shown to have better coverage in simulation studies. However, when we have a response variable that is skewed, even these relaxed assumptions may not be valid, and use of these intervals might therefore result in poor coverage. We propose a new interval (MATA-SBoot) which uses a parametric bootstrap approach to *estimate* the distribution of the studentized estimate for each model, when that model is true. This method only requires that the studentized estimate from each model is approximately pivotal, an assumption that will often be true in practice, even for skewed data. We illustrate use of this new interval in the analysis of a three-factor marine global change experiment in which the response variable is assumed to have a lognormal distribution. We also perform a simulation study, based on the example, to compare the lower and upper error rates of this interval with those for existing methods. The results suggest that the MATA-SBoot interval can provide better error rates than existing intervals when we have skewed data, particularly for the upper error rate when the sample size is small.

## Introduction

It is well known that calculation of a confidence interval after selection of a best model ignores model uncertainty and can lead to the interval having poor coverage [1–5]. A simple alternative is to use an interval based on the full model. In settings where this model provides a good approximation to the “truth”, this will often lead to error rates close to the required levels. Even in these settings, a simpler model may provide a narrower interval with good coverage properties. However, if the data are used to both select a model and to estimate its parameters, the coverage rate can often be much lower than desired. Model-averaging offers a compromise between these two types of intervals, in that we might expect it to lead to a narrower interval than the full model, whilst providing better coverage than an interval based on a single best model [6, 7].

Recently, progress has been made in assessing the theoretical properties of model-averaging, both in terms of optimal weights and construction of confidence intervals. While these results are generally limited to simple settings [8] or rely on asymptotics [9], they provide some insight into the development and understanding of the properties of model-averaged intervals. To complement this work, simulation studies like the one used in this paper are helpful in evaluating the properties of different methods for small sample sizes [4, 7, 10, 11].

Model-averaging is appropriate when interpretation of the parameter of interest, *θ*, is the same for all models. A common example of such a parameter is the expected value of the response variable for a specified combination of predictor variables. Let *M* be the number of candidate models, and ${\hat{\theta }}_{m}$ be the estimate of *θ* from model *m*. In the frequentist setting, the model-averaged estimate of *θ* is a weighted mean of the estimates from the individual models, given by

$$\begin{array}{c} \bar{\theta }=\sum_{m=1}^{M}w_{m}{\hat{\theta }}_{m}, \end{array}$$ (1)

where *w*_*m*_ is the weight for model *m*, with *w*_*m*_ ≥ 0 and $\sum_{m=1}^{M}w_{m}=1$. There are a number of different methods for selecting the model weights. For the rest of the paper, we consider AIC weights, given by

$$\begin{array}{c} w_{m}\propto \exp(-{\text{AIC}}_{m}/2), \end{array}$$ (2)

where AIC_*m*_ is the AIC value for model *m*. In model selection, AIC tends to select larger models than other information criteria, such as AICc or BIC. It is therefore a natural choice in settings where it is reasonable to assume that the full model is closest to “truth”, as in a designed experiment. Previous studies of model-averaged confidence intervals, both theoretical and simulation-based, have also suggested that use of AIC weights is preferable to those based on AICc or BIC [7, 11–13].

Throughout the paper we will refer to the studentized versions of $\bar{\theta }$ and ${\hat{\theta }}_{m}$ as *T* and *T*_*m*_ respectively, i.e.

$$\begin{array}{c} T=\frac{\bar{\theta }-\theta }{\sqrt{\hat{V}(\bar{\theta })}}\;\text{and}\;T_{m}=\frac{{\hat{\theta }}_{m}-\theta }{\sqrt{\hat{V}({\hat{\theta }}_{m})}}\;\left(m=1,\ldots ,M\right). \end{array}$$ (3)

One approach to calculating a model-averaged confidence interval is to use a Wald interval based on $\bar{\theta }$. This involves the assumption that *T* has a *N*(0, 1) distribution [6]. This Wald interval has been used in a wide range of application areas [14–24]. We will refer to this interval as the Model-Averaged Wald Interval (MA-Wald).

Recently, [11] proposed a model-averaged tail area (MATA-Wald) interval which involves calculating a weighted average over the models of lower or upper tail areas of the distribution of *T*_*m*_ when model *m* is true. This involves assuming that *T*_*m*_ in Eq (3) has a *N*(0, 1) or *t*-distribution when model *m* is true. In the context of normal linear regression, the *t*-distribution version of the MATA-Wald interval has been shown to perform better than the MA-Wald interval [11].

Although use of the MA-Wald or the MATA-Wald interval will often be preferable to one based on the full model or on a best model [4, 7], they will clearly not perform well if each *T*_*m*_ is skewed. This might occur when we have a response variable that is skewed, for several reasons. First, the distribution of each ${\hat{\theta }}_{m}$ may be non-normal. Second, each ${\hat{\theta }}_{m}$ and its estimated standard error may be positively correlated [25]. Finally, the estimated standard error of each ${\hat{\theta }}_{m}$ may be more variable than assumed. If the response variable is positively skewed these effects can lead to both *T* and *T*_*m*_ being negatively skewed, which will cause the upper confidence limit to be too low and the upper error rate to be too high.

To overcome these problems, a studentized-bootstrap approach can be used to *estimate* the distribution of *T*_*m*_ when model *m* is true. This involves the less-stringent requirement that each *T*_*m*_ is approximately pivotal when model *m* is true. This will often be a reasonable assumption, even for skewed data. We therefore extend the MATA-Wald interval using a parametric studentized bootstrap, and refer to this as the studentized-bootstrap model-averaged tail area (MATA-SBoot) interval.

The use of bootstrapping in model-averaging was discussed by [6], who considered use of a model-averaged parametric percentile bootstrap (PB) interval. This involves generating bootstrap samples from a fitted model, typically the full model. For each bootstrap sample, the best model is selected and this provides an estimate, ${\hat{\theta }}^{*}$. The PB interval is then given by the appropriate percentiles of ${\hat{\theta }}^{*}$. Use of this interval on real data was considered by [6] and [26], but its coverage properties are not well known. In the single-model setting, the percentile bootstrap is known to be first-order accurate, whereas the studentized bootstrap is second-order accurate [27]. We would therefore expect the MATA-SBoot interval to perform better than the PB interval.

The outline of the paper is as follows. First, we describe the MA-Wald, MATA-Wald, and PB intervals, and introduce the MATA-SBoot interval. We then demonstrate use of the MATA-SBoot interval in a real-life setting that involves using a lognormal model to analyse a three-factor experiment designed to assess the effects of global change on a coralline algae. We use a simulation study based on this example to compare the new MATA-SBoot interval to existing methods including the Wald interval from the full model, which we refer to as Full-Wald, and finish with a discussion and suggestions for further research.

## Methods

As in the single-model setting, we might transform the parameter of interest before model averaging, in order to better satisfy the assumptions associated with a particular method. For example, in the context of logistic regression we might calculate a model-averaged confidence interval for a probability *π* by back-transforming the corresponding interval for logit (*π*). We return to this point when we use a log-transformation in both the example and the simulation study.

### MA-Wald interval

The MA-Wald interval was proposed by [3], and is given by

$$\begin{array}{c} \bar{\theta }\pm z_{1-\alpha }\sqrt{\hat{V}(\bar{\theta })}, \end{array}$$ (4)

where 100 (1 − 2*α*)% is the nominal coverage,

$$\begin{array}{c} \hat{V}(\bar{\theta })=\sum_{m=1}^{M}w_{m}\{(\frac{t_{v_{m},1-\alpha }}{z_{1-\alpha }}{)}^{2}\hat{V}({\hat{\theta }}_{m})+({\hat{\theta }}_{m}-\bar{\theta }{)}^{2}\}, \end{array}$$ (5)

$V({\hat{\theta }}_{m})$ is the variance of ${\hat{\theta }}_{m}$ *conditional* upon model *m* being true (estimated in the usual way after fitting model *m*), $t_{v_{m},1-\alpha }$ is the 100 (1 − *α*)th percentile of the *t*-distribution with *ν*_*m*_ degrees of freedom, *ν*_*m*_ is the residual degrees of freedom associated with model *m*, and *z*_1−*α*_ is the 100 (1 − *α*)th percentile of the *N* (0, 1) distribution [3]. Use of the ratio $t_{v_{m},1-\alpha /z_{1-\alpha }}$ in Eq (5) is motivated by a desire to allow for differences between models in the uncertainty associated with $\hat{V}({\hat{\theta }}_{m})$.

This interval is based on the assumption that the sampling distribution of $\bar{\theta }$ is approximately normal [6]. This assumption is unlikely to be satisfied due to the randomness of the weights, and reliable estimation of the standard error of $\bar{\theta }$ is also difficult [9]. One motivation for the estimate in Eq (5) is that it can be regarded as a frequentist analogue of the variance of a model-averaged posterior distribution for *θ* [3]. As mentioned in the Introduction, *T* will often be negatively skewed when the response variable is positively skewed, leading to this interval having poor coverage.

An alternative Wald interval was proposed by [9]; as this does not have any advantages over the Wald interval from the full model [8, 28], we do not consider it further.

### MATA-Wald interval

The MATA-Wald interval is based on a Wald interval obtained from each model [11]. The *t*-version of the 100 (1 − 2*α*)% MATA-Wald interval [*θ*_*L*_, *θ*_*U*_] is obtained by solving the equations

$$\begin{array}{c} \sum_{m=1}^{M}w_{m}Pr(T_{\nu_{m}}\leq t_{L,m})=\sum_{m=1}^{M}w_{m}Pr(T_{\nu_{m}}\geq t_{U,m})=\alpha , \end{array}$$ (6)

where $T_{\nu_{m}}$ has a *t*-distribution with *ν*_*m*_ degrees of freedom,

$$\begin{array}{c} \;t_{L,m}=\frac{{\hat{\theta }}_{m}-\theta_{U}}{\sqrt{\hat{V}({\hat{\theta }}_{m})}},\;\text{and}\;t_{U,m}=\frac{{\hat{\theta }}_{m}-\theta_{L}}{\sqrt{\hat{V}({\hat{\theta }}_{m})}}. \end{array}$$

Use of this interval is based on the assumption that *T*_*m*_ in Eq (3) has a *t*-distribution with *ν*_*m*_ degrees of freedom when model *m* is true. This assumption will be exact when we are averaging over a set of normal linear models, and may be a reasonable approximation in other settings. In general, for likelihood-based models it is common practice to assume that the sample size is large enough for *T*_*m*_ to have a *N* (0, 1) distribution when model *m* is true. This leads to the *z*-version of the MATA-Wald interval, in which each $T_{\nu_{m}}$ in Eq (6) is replaced by *Z* ∼ *N* (0, 1). Unlike the *t*-version, this makes no allowance for the uncertainty associated with $\hat{V}({\hat{\theta }}_{m})$, which will clearly be undesirable if the sample size is small. The *t*-version of the MATA-Wald interval is therefore likely to be generally more reliable, and will always have a higher coverage rate than the *z*-version.

### Percentile bootstrap interval

This method involves generating *B* bootstrap samples from one of the fitted models, and for each sample selecting the best model according to some criterion. When applying this method in the example and the simulation study, we use AIC to select the best model. The best model for each bootstrap sample provides an estimate ${\hat{\theta }}^{*}$. The 100 (1 − 2*α*)% PB interval is then given by the 100*α*^th^ and 100 (1 − *α*)^th^ percentiles of the distribution of ${\hat{\theta }}^{*}$ over all bootstrap samples.

If the models are nested, as in a factorial experiment, it is natural to use the fitted full model to generate the bootstrap samples, as we expect this to provide a good approximation to the “truth”. In our simulation study, we therefore generate bootstrap samples in this manner. In related work, [29] recommended that bootstrapping should generally be from the full model.

### MATA-SBoot interval

In the single-model setting, a parametric studentized bootstrap interval is given by

$$\begin{array}{c} {}[\hat{\theta }-t_{U}^{*}\sqrt{\hat{V}(\hat{\theta })},\;\hat{\theta }-t_{L}^{*}\sqrt{\hat{V}(\hat{\theta })}], \end{array}$$

where $t_{L}^{*}$ and $t_{U}^{*}$ are the 100*α*^th^ and 100(1 − *α*)^th^ percentiles of the distribution of

$$\begin{array}{c} T^{*}=\frac{{\hat{\theta }}^{*}-\hat{\theta }}{\sqrt{\hat{V}({\hat{\theta }}^{*})}}, \end{array}$$ (7)

and ${\hat{\theta }}^{*}$ is the estimate of *θ* obtained from a bootstrap sample generated from the fitted model. Suppose [*θ*_*L*_, *θ*_*U*_] denotes the resulting interval. The limits of this interval satisfy the equations

$$\begin{array}{c} Pr(T^{*}\leq t_{L}^{*})=Pr(T^{*}\geq t_{U}^{*})=\alpha , \end{array}$$

where T* is given by Eq (7),

$$\begin{array}{c} t_{L}^{*}=\frac{\hat{\theta }-\theta_{U}}{\sqrt{\hat{V}(\hat{\theta })}}\;\text{and}\;t_{U}^{*}=\frac{\hat{\theta }-\theta_{L}}{\sqrt{\hat{V}(\hat{\theta })}}. \end{array}$$

By analogy, in the multi-model setting the MATA-SBoot interval [*θ*_*L*_, *θ*_*U*_] is obtained by solving the equations

$$\begin{array}{c} \sum_{m=1}^{M}w_{m}Pr(T_{m}^{*}\leq t_{L,m}^{*})=\sum_{m=1}^{M}w_{m}Pr(T_{m}^{*}\geq t_{U,m}^{*})=\alpha , \end{array}$$ (8)

where

$$\begin{array}{c} T_{m}^{*}=\frac{{\hat{\theta }}_{m}^{*}-{\hat{\theta }}_{m}}{\sqrt{\hat{V}({\hat{\theta }}_{m}^{*})}},\;t_{L,m}^{*}=\frac{{\hat{\theta }}_{m}-\theta_{U}}{\sqrt{\hat{V}({\hat{\theta }}_{m})}},\;t_{U,m}^{*}=\frac{{\hat{\theta }}_{m}-\theta_{L}}{\sqrt{\hat{V}({\hat{\theta }}_{m})}}, \end{array}$$

and ${\hat{\theta }}_{m}^{*}$ is the estimate of *θ* obtained from fitting model *m* to that bootstrap sample. The probabilities in Eq (8) are estimated from the bootstrap distribution of $T_{m}^{*}$, based on *B* bootstrap samples generated from the fitted version of model *m*.

Use of the bootstrap in this way avoids the need to assume a parametric distribution for *T*_*m*_. We need only require that *T*_*m*_ be approximately pivotal when model *m* is true, an assumption that will be reasonable in many settings [30, 31].

When *T*_*m*_ has a *N* (0, 1) or *t*-distribution, the MATA-SBoot interval will be identical to the corresponding MATA-Wald interval, as long as *B* is chosen to be sufficiently large. We would therefore expect the MATA-SBoot interval to perform at least as well as the two versions of the MATA-Wald interval.

### A factorial design example

Ocean acidification is the process of increasing absorption of anthropogenically-derived CO_2_ by surface seawater [32, 33]. This has negative repercussions for calcareous species, altering growth and calcification rates [34]. Metabolic processes have the potential to modulate the effects of ocean acidification, e.g. photosynthetic uptake of CO_2_ by macroalgae could increase pH back to current levels in large macroalgal forests [35], or at the surface of the macroalga [36, 37]. This has been shown to alleviate the negative effects of ocean acidification for species capable of raising seawater pH [38].

In multi-stressor global change experiments the importance or existence of interactions is generally unknown, so it is not always clear which statistical model should be used to make predictions about physiological responses. While numerous studies have attempted to answer this question (e.g. the meta-analysis in [39]), testing for interactions and then using the selected model to make predictions is precisely the setting that is known to result in poor error rates. Recently, [40] used the MA-Wald interval in Eq (4) to make predictions in a global change experiment, the first example we know of model-averaging being used in this important research area. It is therefore of interest to assess whether there is a better choice of interval.

In this example we use data originally presented in [36] to illustrate the use of model averaging in an investigation of the effect of assemblages of upright and crustose coralline algae to modify their local environment within and immediately above their canopies. Several response variables were measured; our choice of surface hydronium ion concentration ([H_3_O^+^], standardized by bulk concentration) is purely for illustration. In a unidirectional flume, bulk seawater *pH* (ambient pH 8.00, and simulated ocean acidification pH 7.65), *irradiance* (darkness and photosynthetically saturating light), and the effect of water *velocity* (0.015 and 0.040 m s^-1^) were tested on hydronium ion gradients using a 2^3^ factorial design with five replicates.

We focus on estimation of the mean hydronium ion concentration for each of the eight combinations of the factor levels, which we denote as *θ*_*ijk*_ (*i*, *j*, *k* = 1, 2). Thus *θ*_*ijk*_ ≡ E(*Y*_*ijkl*_), where *Y*_*ijkl*_ is the hydronium ion concentration for replicate *l* associated with treatment combination (*i*, *j*, *k*) (*l* = 1, …, *r*). In the example we have *r* = 5, while in the simulation study we consider a range of values for *r*. We assumed the following lognormal model for *Y*_*ijkl*_

$$\begin{array}{c} \log(Y_{ijkl})=\mu_{ijk}+\varepsilon_{ijkl}, \end{array}$$ (9)

$$\begin{array}{c} \mu_{ijk}=\mu +\alpha_{i}+\beta_{j}+\gamma_{k}+\alpha \beta_{ij}+\alpha \gamma_{ik}+\beta \gamma_{jk}+\alpha \beta \gamma_{ijk}, \end{array}$$ (10)

where *μ* is the overall effect, {*α*_*i*_, *β*_*j*_, *γ*_*k*_} are the main effects, {*αβ*_*ij*_, *αγ*_*jk*_, *βγ*_*jk*_} are the two-way interactions, *αβγ*_*ijk*_ is the three-way interaction, and *ε*_*ijkl*_ ∼ *N*(0, *σ*^2^). In the context of this study, *μ*_*ijk*_ is proportional to the mean surface pH of the algae for treatment combination (*i*, *j*, *k*).

We obtained a confidence interval for *θ*_*ijk*_ by back-transformation of the interval for *η*_*ijk*_ ≡ log (*θ*_*ijk*_) = *μ*_*ijk*_ + *σ*^2^/2. The estimate of *η*_*ijk*_ from model *m* is given by

$$\begin{array}{c} {\hat{\eta }}_{ijk,m}={\hat{\mu }}_{ijk,m}+\frac{{\hat{\sigma }}_{m}^{2}}{2}, \end{array}$$ (11)

where ${\hat{\mu }}_{ijk,m}$ is the mean for combination (*i*, *j*, *k*) on the log-scale, and ${\hat{\sigma }}_{m}^{2}$ is the residual mean square from an analysis of variance on this scale. An unbiased estimate of the variance of ${\hat{\eta }}_{ijk,m}$ is given by

$$\begin{array}{c} \hat{V}({\hat{\eta }}_{ijk,m})=\frac{{\hat{\sigma }}_{m}^{2}}{r}+\frac{({\hat{\sigma }}_{m}^{2}{)}^{2}}{2(\nu_{m}+2)}. \end{array}$$ (12)

The expressions for ${\hat{\eta }}_{ijk,m}$ and $\hat{V}({\hat{\eta }}_{ijk,m})$ both involve ${\hat{\sigma }}_{m}^{2}$. When model *m* is true, these two estimates will therefore be positively correlated. In addition, ${\hat{\eta }}_{ijk,m}$ will have a non-normal distribution, and $\nu_{m}\hat{V}({\hat{\eta }}_{ijk,m})/V({\hat{\eta }}_{ijk,m})$ will not have a $\chi_{\nu_{m}}^{2}$ distribution. These effects will mean that the assumptions underlying the MA-Wald interval and both versions of the MATA-Wald interval are invalid. The distribution of both *T* and *T*_*m*_ will then be negatively skewed, leading to the corresponding interval having an upper limit that is too low, and consequently an upper error rate that is too high. All three of the above effects will be more noticeable for smaller values of *r*, and for larger values of *σ*^2^. For the MA-Wald interval there is the additional issue that the model weights are estimated, rather than fixed, and ${\bar{\eta }}_{ijk}=\sum_{m=1}^{M}w_{m}{\hat{\eta }}_{ijk,m}$ may then have a non-normal distribution even if each ${\hat{\eta }}_{ijk,m}$ is close to normal (as they would be if each *ν*_*m*_ were large).

Model-averaging was performed using the set of all possible models. As usual, interaction terms were included only if lower-order terms were also in the model. The AIC weights showed non-negligible support for several models (Table 1). For each *θ*_*ijk*_, we calculated the MA-Wald interval, both versions of the MATA-Wald interval, the percentile bootstrap interval, and the MATA-SBoot interval. We also calculated a Wald interval from the full model (Full-Wald), which is equivalent to using the MA-Wald interval, or the *t*-version of the MATA-Wald interval, with all the weight given to the full model.

**Table 1:** AIC model weights obtained when modelling hydronium ion concentrations. The main effects of pH, irradiance and water velocity are denoted by P, I and V, respectively; PI, IV and PV denote the corresponding two-way interactions and PIV is the three-way interaction.

Model	AIC weight
Null	0.000
P	0.000
I	0.000
V	0.000
P+I	0.028
P+V	0.000
I+V	0.000
P+I+V	0.013
P+I +PI	0.325
P+V+PV	0.000
I+V+IV	0.000
P+I+V+PI	0.150
P+I+V+PV	0.005
P+I+V+IV	0.017
P+I+V+PI+PV	0.057
P+I+V+PI+IV	0.263
P+I+V+PV+IV	0.006
P+I+V+PI+PV+IV	0.100
P+I+V+PI+PV+IV+PIV	0.038

**Fig 1:**
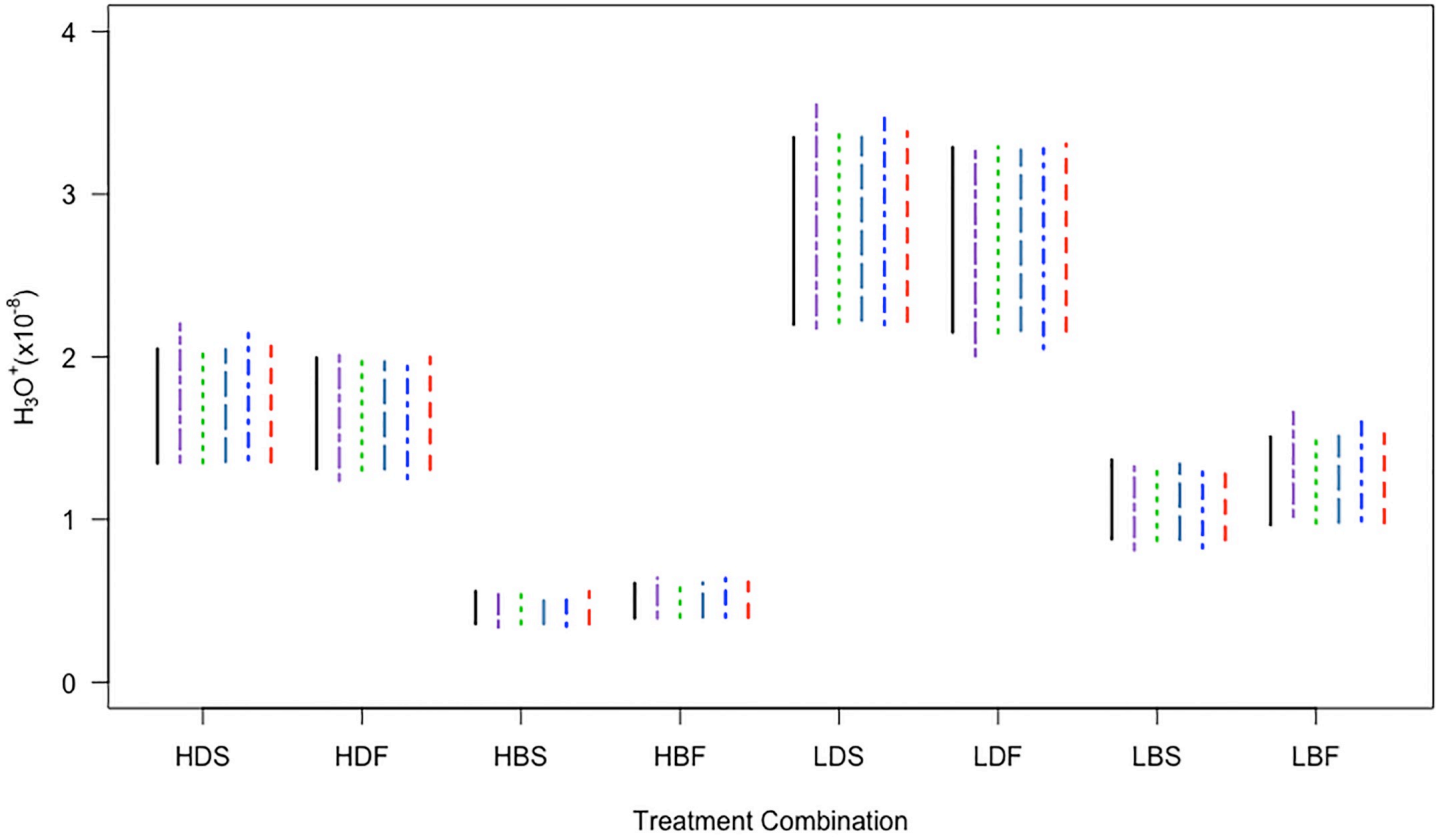
Model-averaged confidence intervals for mean hydronium ion concentration. The intervals are labelled as follows: Full-Wald (long dash-dotted purple line), MA-Wald (solid black line), *t*-version of MATA-Wald (dotted green line), *z*-version of MATA-Wald (long dashed steelblue line), percentile bootstrap (dash-dotted blue line), and MATA-SBoot (dashed red line). The treatment combination denotes the level of each of three factors: pH (H = 8.00, L = 7.65), irradiance (D = darkness, B = photosynthetically saturating light), and velocity (S = 0.015 m s^-1^, F = 0.040 m s^-1^).

For each of the eight combinations of factor levels, the six intervals were broadly similar, the main difference being that the Full-Wald and the percentile interval were generally wider (Fig 1). Although the intervals in this example are similar, we would expect the MATA-Wald and MATA-SBoot intervals to perform quite differently when the sample size is small, as *T*_*m*_ will then be more skewed. We therefore consider a range of sample sizes in the simulation study.

### Simulations

We carried out a simulation study in order to compare the six types of interval. We considered the same setting as the example in Section 3, namely a 2^3^ factorial experiment. The data were generated using the lognormal model in Eqs (9) and (10). As the performance of an interval will not be influenced by the value of *μ*, we set *μ* = 0. We set *σ*^2^ = 1 as this corresponds to a lognormal distribution that is clearly skewed, with a skewness coefficient of 6.2. We return to the choice of *σ*^2^ when discussing the results.

In order to broaden the conclusions of the study, a different set of parameter values was generated for each simulation run, as in [7]. Thus each main effect and interaction was specified as having a “magnitude” of 2 (High), 1 (Medium) or 0.1 (Low). The corresponding parameter value was then selected from a normal distribution with mean zero and standard deviation equal to this magnitude. The three magnitudes were chosen to be greater than, the same as, or less than *σ*^2^. As we usually expect main effects to be at least as large as two-way interactions, which in turn will often be at least as large as three-way interactions, we chose the following ten scenarios: LLL, MLL, HLL, MML, HML, MMM, HMM, HHL, HHM, HHH, where, for example, HML is a scenario in which the main effects, two-way interactions and three-way interaction have high, medium and low magnitudes respectively.

To assess the performance of each interval for various levels of replication, we considered *r* = 2, 5 and 50. The choice *r* = 2 represents the lowest possible sample size for this type of study, corresponding to the greatest skewness of *T* and *T*_*m*_ (for fixed *σ*^2^). The choice *r* = 50 is unlikely to be used in practice, and was included solely to check for asymptotic convergence of the methods. We used 10^5^ simulations for each of the ten scenarios, as this allowed us to achieve binomial standard errors for the lower and upper error rates of approximately 0.3%. For the bootstrap-based intervals, we used *B* = 9999. As for the real data, we first calculated a confidence interval for *η*_*ijk*_ and back-transformed it to obtain an interval for *θ*_*ijk*_ ≡ exp (*η*_*ijk*_), which we denote as $\left[\theta_{ijk}^{L},\theta_{ijk}^{U}\right]$. Model-averaging was performed over all 19 possible models.

The performance of each interval was summarised by its mean, over the eight combinations of factor levels, of the lower and upper error rate. We also calculated the mean lower and upper relative half-widths for each treatment combination, and averaged these over the eight combinations, where the relative lower and upper half-width are defined as $(\theta_{ijk}^{L}-\theta_{ijk})/\theta_{ijk}$ and $(\theta_{ijk}^{U}-\theta_{ijk})/\theta_{ijk}$ respectively. All calculations were implemented in R Version 3.4.2 [41], and the solutions to Eqs (6) and (8) were found using the *uniroot* function. We also include example code in Supplementary Information (S1 File) demonstrating calculation of the MATA-SBoot interval for a single dataset, and note that functions to calculate the MATA-Wald interval are available in the MATA library of R [11].

## Results

**Fig 2:**
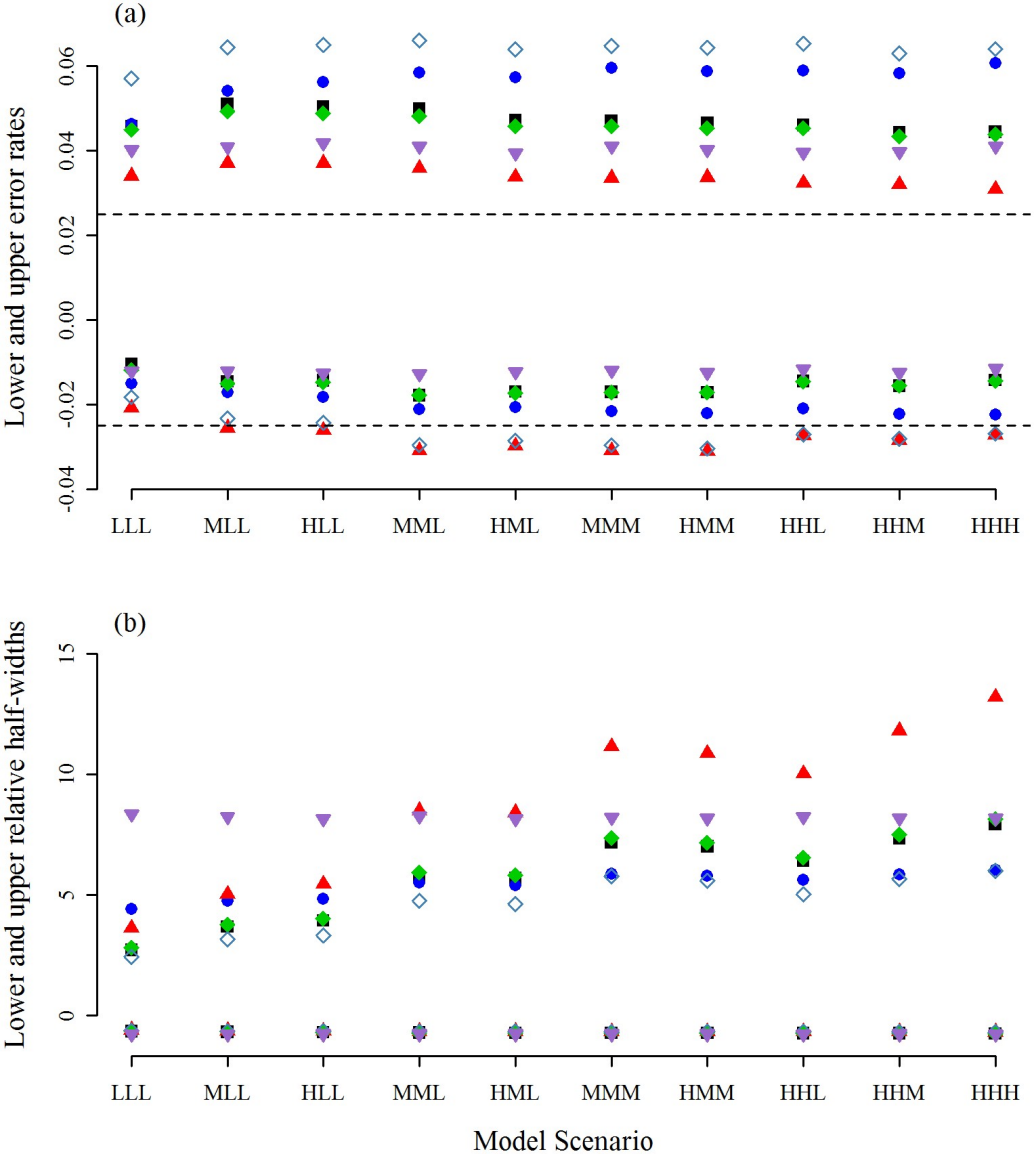
Error rates and relative half-widths when *r* = 2. The results obtained using the Full-Wald (down-pointing purple triangle), the MA-Wald (black square), the *t*-version of the MATA-Wald interval (green diamond), the *z*-version of the MATA-Wald interval (unfilled steelblue diamond), the percentile interval (blue circle), and the MATA-SBoot interval (up-pointing red triangle). For simplicity, the lower error rates are plotted on the negative axis. The nominal rate is shown as a dashed line.

**Fig 3:**
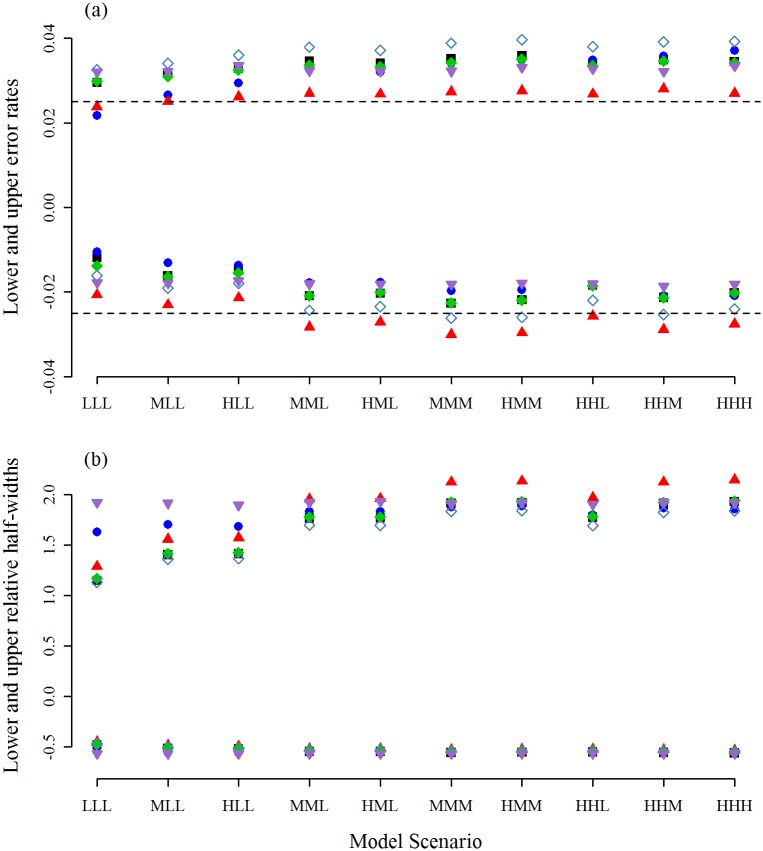
Error rates and relative half-widths when *r* = 5. The results obtained using the Full-Wald (down-pointing purple triangle), the MA-Wald (black square), the *t*-version of the MATA-Wald interval (green diamond), the *z*-version of the MATA-Wald interval (unfilled steelblue diamond), the percentile interval (blue circle), and the MATA-SBoot interval (up-pointing red triangle). For simplicity, the lower error rates are plotted on the negative axis. The nominal rate is shown as a dashed line.

**Fig 4:**
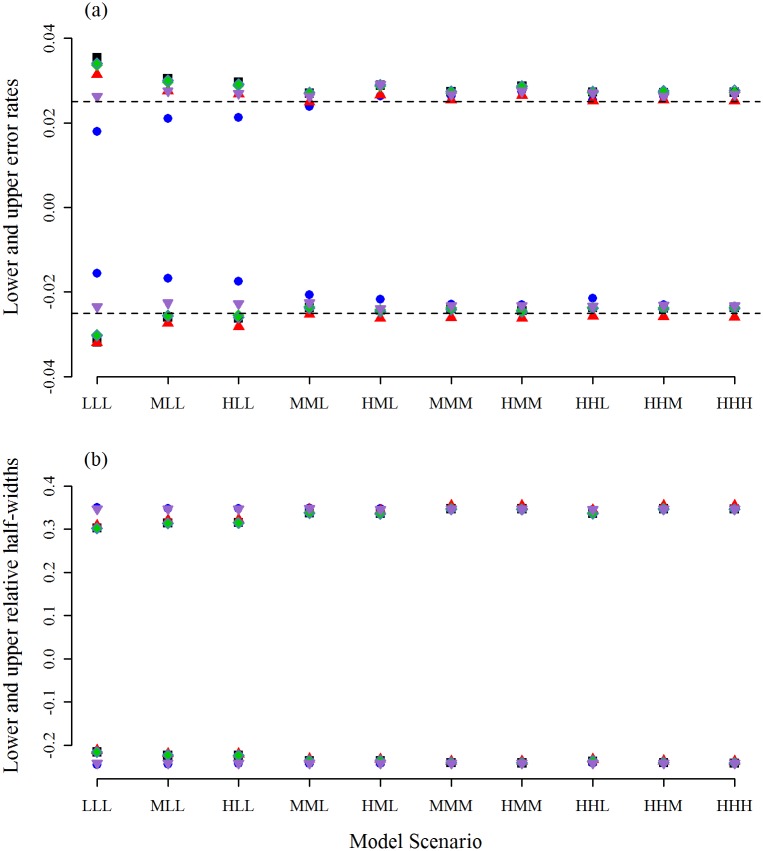
Error rates and relative half-widths when *r* = 50. The results obtained using the Full-Wald (down-pointing purple triangle), the MA-Wald (black square), the *t*-version of the MATA-Wald interval (green diamond), the *z*-version of the MATA-Wald interval (unfilled steelblue diamond), the percentile interval (blue circle), and the MATA-SBoot interval (up-pointing red triangle). For simplicity, the lower error rates are plotted on the negative axis. The nominal rate is shown as a dashed line.

The clearest difference between the methods are for the upper confidence limit, with the MATA-SBoot interval generally having an upper error rate that is closest to the nominal level (Figs 2 to 4). This improvement in the upper error rate is most marked for *r* = 2, as we expected. The MATA-SBoot interval also provided a lower error rate that was close to the nominal level. Because the MATA-SBoot increases its width to account for skewness, it was always wider than the MA-Wald and MATA-Wald intervals and usually wider than the PB interval. Interestingly, for the LLL, MLL, and HLL scenarios, the improvement was achieved with little increase in the upper half-width relative to other model-averaging techniques, while substantially outperforming the Wald-Full interval. However, for scenarios with higher magnitude effects and few replicates (Fig 2), the increased width from skewness led to substantially wider upper half-widths for MATA-SBoot intervals relative to the others.

The *z*-version of the MATA-Wald interval performed worst of all the model-averaging methods on the upper error rate, but very well on the lower error rate, presumably because any skewness to the left still allowed the right-hand tail *T*_*m*_ to be similar to that for a *N*(0, 1) distribution. The generally superior performance of the *t*-version over the *z*-version of the MATA-Wald interval is due to it making some allowance for the uncertainty associated with $\hat{V}({\hat{\theta }}_{m})$, the difference being largest when *r* is small. The MA-Wald interval incorporates the ratio $t_{v_{m},1-\alpha /z_{1-\alpha }}$ as a means of allowing for this uncertainty, which provides another reason for its performance being similar to that for the *t*-version of the MATA-Wald interval.

Unlike the model-averaged intervals, the interval widths for Full-Wald do not vary across the different simulation scenarios. Particularly, they do not take advantage of the smaller widths possible through model-averaging when weight is placed on reduced models. Consequently, the intervals are wider or equal to the intervals from MA-Wald, MATA-Wald, or PB intervals. However, the increased upper error rates of the Full-Wald interval also produce narrower upper half-widths than MATA-SBoot for scenarios with large magnitude effects (Fig 2).

All methods except the PB interval had approximately the same error rates for *r* = 50 (Fig 4). The PB interval is the only method that does not involve studentization, and suffers from being unnecessarily wide when *r* = 50, with the upper and lower error rates both being less than required, especially for the LLL, MLL, and HLL scenarios.

## Discussion

We have focussed on using a bootstrap-based method to construct a model-averaged confidence interval. Bootstrapping can also be used to select model weights [26, 42–44]. For example, we might choose *w*_*m*_ to be the proportion of times over all bootstrap samples that model *m* is selected as the best model. This type of weight is implicit in calculation of the PB interval, as well as in the use of bagging to calculate a model-averaged point estimate, a technique that has been used widely in machine learning [45, 46]. In our simulation study, when calculating the PB interval, we found that using AIC to select the best model led to this weight being very similar to the AIC weight in Eq (2), in agreement with the results of [6].

In our simulation setting, the MATA-SBoot interval provided a consistent improvement over existing methods when the sample size was small enough for *T* and each *T*_*m*_ to be skewed. Our results suggest that this interval has a better error rate than the other methods for small *r*, while maintaining good error rates and small relative half-widths for large *r*. This difference is most marked for the upper error rate, as *T* and *T*_*m*_ are both negatively skewed.

When it is reasonable to assume that *T*_*m*_ has a *N* (0, 1) or *t*-distribution, the MATA-SBoot interval is equivalent to the relevant MATA-Wald interval, as long as *B* is large enough. The MATA-Wald interval has the advantage of being computational quicker, which might be important when the number of models is large or some of the models are complex.

We chose to set *σ*^2^ = 1 in the simulations, which corresponds to a skewness coefficient of 6.2. If we had used *σ*^2^ < 1 there would have been less skewness and the results for the MATA-Wald and MATA-SBoot intervals would be more similar. Conversely, if had set *σ*^2^ > 1, there would have been more skewness and the benefits of using the MATA-SBoot interval would be even clearer.

The MATA-SBoot interval will obviously not perform well if the studentized bootstrap itself is prone to problems, such as when the standard error of ${\hat{\theta }}_{m}$ is poorly estimated and/or *T*_*m*_ is clearly not pivotal. This caveat is similar to that given by [11] for the MATA-Wald interval [8]. Likewise, in general the MATA approach to constructing a model-averaged confidence interval does not guarantee that the coverage will be exactly as desired, even if the distributional assumptions underlying its use are met [8, 12, 13].

In the simulations the response variable was known to have a lognormal distribution. This allowed us to use an unbiased estimate of the standard error of each ${\hat{\theta }}_{m}$ in both versions of the MATA-Wald interval and in the MATA-SBoot interval. In some settings, the true distribution of the response variable may differ from what we assume. We would expect the MATA-SBoot interval to be more robust than the other methods to such misspecification, as long as *T*_*m*_ is approximately pivotal.

The parameter of interest in the example and simulation study was the population mean (for each treatment combination). With skewed data, we might consider estimation of the population median instead. This would amount to removing the second terms on the right-hand side of both Eqs (11) and (12), leading to ${\hat{\eta }}_{ijk,m}$ and $\hat{V}({\hat{\eta }}_{ijk,m})$ being uncorrelated. The studentized version of ${\hat{\eta }}_{ijk,m}$ would then have a *t*-distribution with *ν*_*m*_ degrees of freedom. In this case, the *t*-version of the MATA-Wald interval and the MATA-SBoot interval would be identical, as long as *B* were chosen to be large enough.

It is difficult to establish theoretical results about the performance of model-averaged confidence intervals, due to the randomness of the model weights. Moreover, model uncertainty will usually arise when the sample size is relatively small, suggesting that asymptotic theory may not be that relevant. We have therefore used simulation to assess the potential benefits of our proposed method. We have considered a range of model-scenarios and used a random-effects generating model in order to make our conclusions more robust. However, as with any simulation study, the conclusions are strictly limited to a particular setting. It would be helpful to assess the performance of the MATA-SBoot interval in other settings, in order to broaden our conclusions.

It is important to note that there exists no unique way to assess the performance of the methods for simulation studies. In our simulation study, we focus on assessing the overall performance of the methods across a number of scenarios. An alternative approach is to compare the minimum coverage of the methods across all possible parameter values, as suggested in [47]. It would be helpful to explore this option in future to further assess the performance of the methods.

For global change studies such as our example, a primary goal is to make predictions under future climate scenarios with an appropriate measure of uncertainty. Use of the MATA-SBoot method can sometimes lead to a narrower confidence interval than one from the full model, whilst maintaining error rates that are generally close to the nominal level, while in other cases the interval must be larger to account for the underlying skewness. Compared to other model-averaging techniques, interval widths are increased due to skewness but, again, this is to maintain approximately the correct level of uncertainty.

## Supporting information

S1 File R code and data for obtaining MATA-SBoot intervals for the hydronium ion example. (R)
